# The Necessity of and Methods for Better Pulp Protection in Filling Teeth

**Published:** 1896-01

**Authors:** J. D. Patterson

**Affiliations:** Kansas City, Mo.


### The Necessity of and Methods for Better Pulp Protection in Filling Teeth.

BY J. D. PATTERSON, D.D.S., KANSAS CITY, MO.

Abstract of a paper read at the American Dental Association, August, 1895.

In considering this subject no reference will be made to pulps which have become exposed or are in a pathological condition from near exposure, but to fairly deep-seated cavities in the teeth of patients under twenty-five years of age, which are usually filled with the prevailing materials without any endeavor to prevent pulp irritation, the operator believing that no considerable trouble will be possible either from the presence of a filling with the property of conducting thermal changes, or from the irritant nature of other filling materials in common use.

In this class of cases every observant practitioner has had his attention directed to the number of pathological conditions arising in after years from death of the pulp. Where there is soon occurring trouble after the insertion of a filling upon a nearly exposed or pathological pulp, the removal of the filling and usual treatment finds immediate cure, but where the pulp death comes from long-continued, generally imperceptible irritation, the danger is greater.

Dr. Patterson presented two cases for the consideation of the society. The first, showed the loss of five teeth and the surrounding process in the mouth of a youug lady, aged twenty, from the death of a pulp under a gold filling in a lateral incisor. The filling was inserted thirteen months before the inflammation began. Some irritation, through thermal changes, was felt after the insertion of the filling, which soon subsided.

When I first saw the case, only four days after the inflammation began, the lateral, central, and two bicuspids could be removed with the thumb and finger, and they were at once removed, as the process in the vicinity of these teeth was stripped of periosteum. When the trouble first showed, the lateral was drilled into ar d mummified pulp remnants were found. Vast quantities of pus followed rapid swelling. The most persistent surgical treatment was instituted, but without avail. The cuspid had to be removed on the sixth day. After extraction of the teeth the inflammation abated somewhat, and after three days more the process, piece after piece, sequestered and was removed. All of this resulting from a small gold filling in the lateral which did not approach the nerve half way through the dentine.

The second case was almost a parallel one, save that the death of the pulp resulted from a cement filling inserted in a central incisor seven years before. Both patients were found to be free from hereditary taint.

As a protective capping gutta-percha stands in the first place. However, it cannot be used save in deep cavities where there is room for a good layer of cement over it upon which to condense gold.

Non-cohesive gold is advocated for deep-seated cavities; for it seems to be less of a conductor of heat and cold than the cohesive gold as condensed with a mallet. The reason of this is because a non-cohesive filling is in no way a cohesive mass; cannot be welded together, and when a cavity is filled with a layer after layer of this gold the thermal shock is conducted along the layers and not down to the bottom of the cavity, for there is no intimate connection between the different particles of the different sheets.

In this material we have an admirable pulp protector. A pad of sufficient thickness made by doubling strips upon each other and burnished over the pulp, has, in my hands, secured the best results.

